# When controversies in null hypothesis significance testing prove to be controversial

**DOI:** 10.1111/aogs.14456

**Published:** 2022-09-12

**Authors:** Philip M. Sedgwick, Anne Hammer, Ulrik Schiøler Kesmodel, Lars Henning Pedersen

**Affiliations:** ^1^ Institute for Medical and Biomedical Education St George's, University of London London UK; ^2^ Department of Obstetrics and Gynecology Gødstrup Hospital Gødstrup Denmark; ^3^ Department of Clinical Medicine Aarhus University Aarhus Denmark; ^4^ Department of Obstetrics and Gynecology Aalborg University Hospital Aalborg Denmark; ^5^ Department of Clinical Medicine Aalborg University Aalborg Denmark; ^6^ Department of Biomedicine Aarhus University Aarhus Denmark; ^7^ Department of Obstetrics and Gynecology Aarhus University Hospital Aarhus Denmark

*Sir*,

We thank Dr. Frane for his Letter to the Editor^1^ in response to our commentary “Current controversies: Null hypothesis significance testing.”^2^

We believe the wording in our commentary regarding the probability of a type I error occurring when 20 hypothesis tests are performed may have been unfortunate. We stated “…when 20 hypothesis tests are performed the probability of a type I error is at least 0.64”. It would have best read “…when 20 independent statistical hypothesis tests are performed the maximum probability of a type I error occurring is approximately 0.64”.^3^

Frane questions the validity of the statement: “Studies with large sample sizes are important because as sample size approaches the population size, the sample estimates have increased accuracy when estimating the population parameters”. This statement regarding the accuracy of sample estimates is undeniably true. As sample sizes approach the population size and more data are collected, then sample estimates will become more accurate in relation to the population parameters that they are estimating. As Frane suggests, the limiting factor is the absolute gain in accuracy which decreases as sample size increases. Nonetheless, we do not agree with the suggestion that “a sample size of 100 does not tend to provide substantially better estimates when it comes from a population of 2000 than when it comes from a population of 2 billion”. Any gain in accuracy is relative and dependent on the clinical situation being researched.

Frane queries the validity of a statement in an article^4^ cited in our commentary. In particular, he claims the author of the cited article incorrectly claimed that as sample size approaches the population size, the type I error rate of each test decreases. Such a claim has no relevance in the context of this commentary, and equally any responses should be directed to the publishing journal itself. Nonetheless, no such claim was made to that effect in the cited article.

We agree with Frane in his statement that “…one statistical misunderstanding often leads to another”. However, we do not believe there are any inappropriate statements or conclusions in this commentary, and therefore no corrections or further clarifications are needed.
